# Dysregulation of the chromatin environment leads to differential alternative splicing as a mechanism of disease in a human model of autism spectrum disorder

**DOI:** 10.1093/hmg/ddad002

**Published:** 2023-01-09

**Authors:** Calvin S Leung, Shoshana J Rosenzweig, Brian Yoon, Nicholas A Marinelli, Ethan W Hollingsworth, Abbie M Maguire, Mara H Cowen, Michael Schmidt, Jaime Imitola, Ece D Gamsiz Uzun, Sofia B Lizarraga

**Affiliations:** Department of Molecular Biology, Cell Biology and Biochemistry, Brown University, Providence, RI 02912, USA; Center for Translational Neuroscience, Carney Institute for Brain Science and Brown Institute for Translational Science (BITS), Brown University, Providence, RI 02912, USA; Center for Computational Molecular Biology, Brown University, Providence, RI 02906, USA; Department of Pathology and Laboratory Medicine, Warren Alpert Medical School of Brown University, Providence, RI 02912, USA; Department of Pathology and Laboratory Medicine, Rhode Island Hospital and Lifespan Academic Medical Center, Providence, RI 02903, USA; Department of Biological Sciences, University of South Carolina, Columbia, SC 29208, USA; Department of Biological Sciences, University of South Carolina, Columbia, SC 29208, USA; UCONN Health Comprehensive Multiple Sclerosis Center, Department of Neurology, University of Connecticut School of Medicine, Farmington, CT 06030, USA; Division of Multiple Sclerosis and Translational Neuroimmunology, Department of Neurology, University of Connecticut School of Medicine, Farmington, CT 06030, USA; Department of Molecular Biology, Cell Biology and Biochemistry, Brown University, Providence, RI 02912, USA; Center for Translational Neuroscience, Carney Institute for Brain Science and Brown Institute for Translational Science (BITS), Brown University, Providence, RI 02912, USA; Department of Biological Sciences, University of South Carolina, Columbia, SC 29208, USA; Department of Molecular Biology, Cell Biology and Biochemistry, Brown University, Providence, RI 02912, USA; Center for Translational Neuroscience, Carney Institute for Brain Science and Brown Institute for Translational Science (BITS), Brown University, Providence, RI 02912, USA; UCONN Health Comprehensive Multiple Sclerosis Center, Department of Neurology, University of Connecticut School of Medicine, Farmington, CT 06030, USA; Division of Multiple Sclerosis and Translational Neuroimmunology, Department of Neurology, University of Connecticut School of Medicine, Farmington, CT 06030, USA; Center for Computational Molecular Biology, Brown University, Providence, RI 02906, USA; Department of Pathology and Laboratory Medicine, Warren Alpert Medical School of Brown University, Providence, RI 02912, USA; Department of Pathology and Laboratory Medicine, Rhode Island Hospital and Lifespan Academic Medical Center, Providence, RI 02903, USA; Department of Molecular Biology, Cell Biology and Biochemistry, Brown University, Providence, RI 02912, USA; Center for Translational Neuroscience, Carney Institute for Brain Science and Brown Institute for Translational Science (BITS), Brown University, Providence, RI 02912, USA

## Abstract

Autism spectrum disorder (ASD) affects 1 in 44 children. Chromatin regulatory proteins are overrepresented among genes that contain high risk variants in ASD. Disruption of the chromatin environment leads to widespread dysregulation of gene expression, which is traditionally thought of as a mechanism of disease pathogenesis associated with ASD. Alternatively, alterations in chromatin dynamics could also lead to dysregulation of alternative splicing, which is understudied as a mechanism of ASD pathogenesis. The anticonvulsant valproic acid (VPA) is a well-known environmental risk factor for ASD that acts as a class I histone deacetylase inhibitor. However, the precise molecular mechanisms underlying defects in human neuronal development associated with exposure to VPA are understudied. To dissect how VPA exposure and subsequent chromatin hyperacetylation influence molecular signatures involved in ASD pathogenesis, we conducted RNA sequencing (RNA-seq) in human cortical neurons that were treated with VPA. We observed that differentially expressed genes (DEGs) were enriched for mRNA splicing, mRNA processing, histone modification and metabolism related gene sets. Furthermore, we observed widespread increases in the number and the type of alternative splicing events. Analysis of differential transcript usage (DTU) showed that exposure to VPA induces extensive alterations in transcript isoform usage across neurodevelopmentally important genes. Finally, we find that DEGs and genes that display DTU overlap with known ASD-risk genes. Altogether, these findings suggest that, in addition to differential gene expression, changes in alternative splicing correlated with alterations in the chromatin environment could act as an additional mechanism of disease in ASD.

## Introduction

Autism spectrum disorder (ASD) is a highly heritable neurodevelopmental disorder with complex genetic etiology (1). Several lines of evidence suggest that gene–environment interaction could influence the risk of developing ASD and contribute to its complex etiology (2,3). Valproic acid (VPA) is an anticonvulsant drug used in the treatment of epilepsy and is a well-characterized environmental risk factor for ASD (4). An increasing number of studies have shown that prenatal exposure to VPA during pregnancy increased the risk of ASD (4–8). Exposure to VPA during pregnancy has also been associated with the fetal valproate syndrome, characterized by a higher incidence of neural tube defects, craniofacial abnormalities, developmental delay, intellectual disability and ASD (9,10). However, mechanistic studies that interrogate the association between VPA exposure and increased ASD risk, or neurodevelopmental defects in humans, are lacking.

Acetylation of histones by histone acetyltransferases (HATs) results in a dispersed or ‘open’ chromatin conformation, allowing physical access for transcription factors to bind to DNA and generally activates transcription (11,12). Conversely, histone deacetylases (HDACs) remove acetyl groups from histones, compacting chromatin and in general are thought to lead to transcriptional repression (12). VPA was identified as a class I selective HDAC inhibitor and has also been found to induce proteasomal degradation of HDAC2 (13,14). VPA’s HDAC inhibition has been shown to be relevant to ASD pathogenesis, because a VPA analog that lacks the HDAC inhibitory function does not cause ASD-like behaviors in mice (15). HDAC inhibition leads to hyperacetylation of histones H3 and H4 and results in transcriptional dysregulation (16,17). In addition to its role in regulating transcription, histone acetylation has also been shown to contribute to the regulation of RNA splicing, an essential process of eukaryotic gene expression (18–20). Increasing evidence suggest that a dysregulated chromatin environment caused by HDAC inhibition modulates alternative splicing (21,22). Therefore, HDAC inhibition by VPA could alter both gene expression and alternative splicing, which suggests a plausible molecular mechanism underlying VPA’s role as an environmental risk factor for ASD. Taken together, these findings suggest the potential involvement of chromatin regulatory mechanisms in the underlying pathology of ASD associated with VPA exposure.

Epidemiological and animal studies suggest a strong association between prenatal exposure to VPA during the first gestational trimester and higher incidence of ASD and birth defects (6,23,24). At this developmental stage, the cerebral cortex is the most rapidly developing structure in the brain. Development of the cerebral cortex requires the careful coordination of progressive complex cellular events such as neuronal progenitor proliferation, neuronal migration, generation of neurons, neuronal morphogenesis and synapse development (25). Even slight alterations in the timing of these processes could impact cortical development and lead to deficits in cognition and behavior (26). The advent of human stem cell derived neural models constitutes an unprecedented opportunity to study the molecular mechanisms underlying the early stages of human brain development that would be otherwise inaccessible (27–29). To investigate the effect of VPA exposure on human neuronal development and identify molecular mechanisms underlying ASD pathogenesis, we used male human induced pluripotent stem cells (iPSCs) of a neuro-typical individual with no known history of neuropsychiatric disorders to derive forebrain cortical excitatory neurons (30). We used a dual-SMAD inhibition protocol to generate cortical excitatory neurons as it recapitulates the temporal sequence in which neurons arise during development (30). Therefore, this approach represents a robust system to analyze transcriptomic changes during early human fetal development (31).

To determine the transcriptomic changes that occur with VPA exposure, we performed RNA-sequencing (RNA-seq) on day 65 iPSC-derived neurons treated with VPA for 24 h on day 64 of neuronal induction. Our analysis revealed 6208 differentially expressed genes (DEGs). Gene set enrichment analysis (GSEA) revealed that these DEGs were enriched for mRNA splicing, mRNA processing, histone modification and metabolism-related gene sets. Remarkably, we identified a distinct signature of differentially spliced genes in neurons treated with VPA that implicates dysregulation of RNA splicing as a mechanism of ASD pathogenesis. Subsequent differential transcript usage (DTU) analysis identified 3848 genes that showed significant DTU events. We find that genes with DTU shared little overlap with DEGs and were enriched for neurodevelopmental disorder-related genes. However, we found that both DEGs and genes that display DTU largely overlap with known ASD risk genes. Considering the large number of chromatin regulators in which high and strong risk variants have been associated with ASD, including histone acetylation modifiers, dysregulation of alternative splicing could be a common mechanism of disease in ASD (32). In fact, transcriptomic studies of different subsets of ASD postmortem brain tissue point to an overrepresentation of differential splicing events in patient samples compared with controls (33,34). However, the extent to which changes in alternative splicing occur at early stages of human neuronal development in relation to specific ASD genetic or environmental risk factors is understudied. Here, we show, for the first time, that exposure to a well-known environmental risk factor for ASD at early stages of human cortical development, leads to changes in gene expression and alternative splicing that may act synergistically as a mechanism underpinning ASD pathogenesis.

## Results

### VPA elicits a distinct molecular signature in human forebrain cortical excitatory neurons

**Figure 1:**
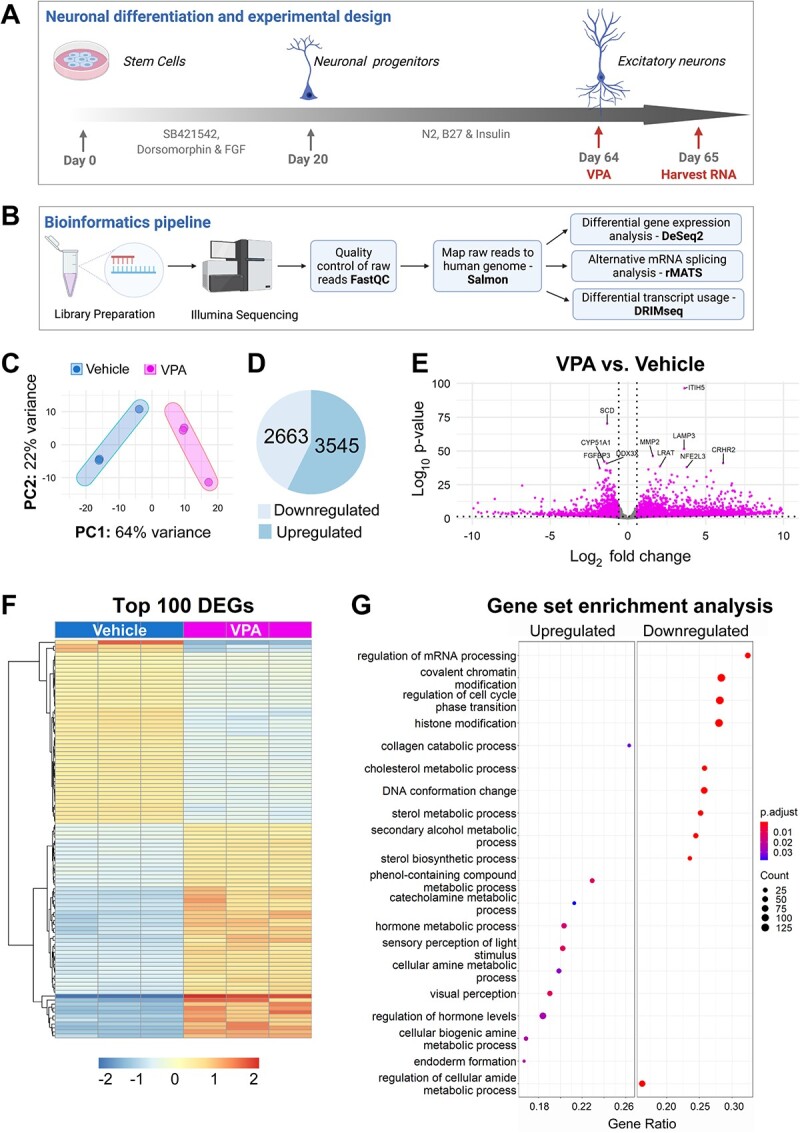
VPA elicits differential gene expression in human cortical excitatory neurons. (**A**) Generation of human cortical excitatory neurons from iPSCs and experimental paradigm. Diagram shows key steps of a dual SMAD inhibition protocol used to generate human forebrain cortical excitatory neurons. Addition of dorsomorphin and SB421542 promotes neural fate by blocking the formation of mesoderm and endoderm. Neurons were allowed to mature for 65 days after the initiation of neuronal induction. On Day 64, VPA (1.2 mM) was added to the neurons for 24 h. RNA was extracted for RNA-seq analysis on Day 65. (**B**) Flow chart of the bioinformatics analysis pipeline. RNA libraries were constructed and processed using an Illumina platform. Quality control of raw reads was performed using FastQC. Trimmed reads were mapped to the human genome (GRCh38) using Salmon. Differential gene expression analysis was performed using DESeq2, alternative mRNA splicing analysis was performed using rMATS and DTU analysis was performed using DRIMSeq. (**C**) PCA of RNA-seq samples of neurons treated with vehicle (blue) or VPA (magenta). To better visualize the closely overlapping points, we used point jittering a visualization method that adds small random variation to the location of each point. (**D**) Pie chart showing significantly upregulated and downregulated DEGs with cutoffs log_2_ fold changes (LFC) > |1.5| and adjusted *P* < 0.05. (**E**) Volcano plot showing indicated changes in LFC gene expression (*x*-axis) and −log_10_  *P* values (*y*-axis) generated from DESeq2 analysis. Vertical dotted lines represent 0.58 LFC (1.5 FC) and the horizontal dotted line represents adjusted *P* = 0.05. Top 10 significant genes based on *P*-value are labeled. (**F)** Heat map representation of differential gene expression. The top 100 DEGs were selected based on their adjusted *P*-values. Each column represents an independent sample that was either treated with vehicle or treated with VPA. Gene count values were plotted, and color coded to represent expression with red indicating high expression levels and blue indicating low expression levels of individual genes. (**G**) Pathway enrichment analysis was performed using GSEA algorithm with Human Molecular Signatures Database (MSigDB). Dot plot displaying GSEA results of upregulated and downregulated DEGs (*P*-value cutoff = 0.05). Top 20 enrichment results are shown.

Weighted gene co-expression analysis studies have identified forebrain cortical neurons as hubs for the expression of genes that have high-risk variants associated with ASD (35). Because ASD has a higher incidence in males than in females, we examined the effect of VPA on male human iPSC-derived forebrain cortical neurons from a neurotypical individual that were differentiated for 65 days (36). iPSCs were differentiated to cortical forebrain excitatory neurons by inhibiting the BMP and TGF-β signaling pathways to induce the forebrain neuroectodermal lineage (Fig. 1A) (30). To uncouple the effect of VPA on neuronal progenitors, we used neurons grown for 65 days as they already start displaying synaptic activity at this stage (28). We isolated high-quality RNA from control and VPA-treated samples that were used to prepare RNA-seq libraries (Supplementary Material, Fig. S1). RNA-seq was performed to determine the genome-wide effects of VPA exposure. We used the Salmon software package (37) to quantify transcript abundance from the RNA-seq reads, allowing for both downstream differential gene expression and DTU analysis (Fig. 1B and Supplementary Material, Table S1). Using the DESeq2 software package, we observed, via principal component analysis (PCA), that the sequenced samples showed distinct clustering of biological replicates by treatment group, suggesting that VPA treatment contributed to a distinct transcriptomic profile compared with untreated neurons (Fig. 1C). We identified statistically significant DEGs with adjusted *P* < 0.05 and FC ≥ |1.5|. We observed widespread and distinct changes in gene expression in the VPA-treated samples compared with control samples with 3545 upregulated and 2663 downregulated DEGs (Fig. 1D–F, and Supplementary Material, Table S2). Next, we conducted quantitative PCR (qPCR) validation on representative upregulated and downregulated DEGs to confirm the RNA-seq results (Supplementary Material, Fig. S2 and Supplementary Material, Table S3).

To begin to define the extent to which there were specific molecular signatures altered in response to VPA, we conducted GSEA of DEGs (Supplementary Material, Table S4). Among the topmost enriched gene categories, we found downregulated DEGs that were implicated in the regulation of mRNA processing, covalent chromatin modification, regulation of cell cycle phase transition and histone modification (Fig. 1G). Interestingly, multiple genes encoding mRNA splicing factors were observed within the regulation of mRNA processing pathway. mRNA splicing factors are involved in the formation of mature mRNA through intron excision and exon ligation of precursor mRNA (pre-mRNA). Additionally, alternative mRNA splicing allows for a single gene to generate an average of three alternatively spliced mRNA isoforms, leading to increased complexity of gene expression and protein function in the cell (38). Individual gene count analysis of representative alternative splicing factors *PTBP1* (log_2_ FC = −0.6018725), *SRSF12* (log_2_ FC = −1.186584437) and *RBFOX2* (log_2_ FC = −0.5403933) showed consistent downregulation of these genes (Supplementary Material, Fig. S3). Downregulation of alternative splicing factors and other mRNA processing genes suggests that VPA may also lead to changes in transcript isoform usage.

In addition to the distinct mRNA processing signature, we identify a chromatin regulatory signature in response to VPA exposure, as genes involved in covalent chromatin modification, histone modification and DNA conformation change pathways were also found to be downregulated. Genes in these pathways include histone methyltransferases, histone demethylases, histone acetyltransferases, histone deacetylases and chromatin remodelers. Representative genes in these categories include: *NSD1* (log_2_ FC = −1.225422657), *KDM5B* (log_2_ FC = −1.038048715), *KAT2B* (log_2_ FC = −1.312303), *HDAC6* (log_2_ FC = −0.631808) and *CHD2* (log_2_ FC = −0.9104716) (Supplementary Material, Fig. S3). Regulation of the chromatin landscape by chromatin modifiers and/or remodelers has been shown to have widespread effects in the control of gene expression. Furthermore, specific chromatin modifications have also been associated with the control of RNA splicing. Thus, downregulation of chromatin modifiers and remodelers in response to VPA treatment could have an additive effect in the control of gene expression and alternative splicing, and exacerbate VPA-related phenotypes.

### Profiling of the alternative mRNA splicing landscape in VPA-treated neurons

**Figure 2:**
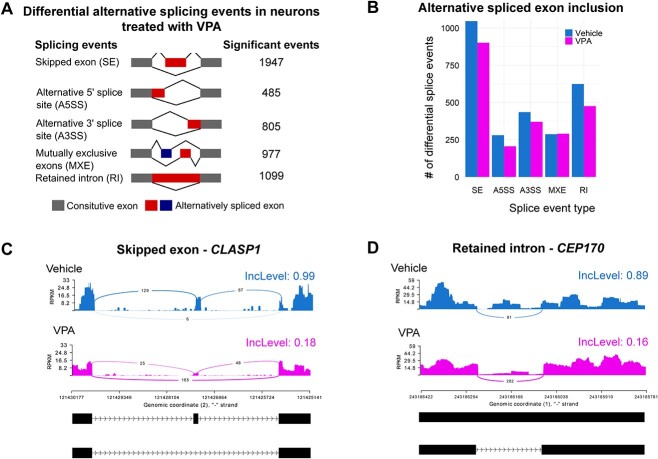
VPA induces widespread changes in alternative mRNA splicing in human neurons. (**A**) Diagram showing the different types of alternative mRNA splicing events identified using rMATS and number of significant events (ΔPercent spliced in (PSI) > 0.1; FDR < 0.05) for each event after filtering (>20 alternative exon counts). (**B**) Bar plot displaying number of statistically significant alternatively spliced exon inclusion events for the different types of alternative mRNA splicing events in neurons treated with vehicle (blue) or VPA (magenta). Three RNA-seq replicates for control and VPA-treated samples were used as input in the rMATS differential alternative splicing analysis software. The rMATS software detected statistically significant changes in alternative splice site usage in the VPA-treated samples versus the control samples. (**C**) Sashimi plot of the gene *CLASP1* showing an SE event. Alternative exon inclusion levels (IncLevel) for vehicle and VPA are indicated. (**D**) Sashimi plot of *CEP170* shows an RI event. Alternative exon inclusion levels (IncLevel) for vehicle and VPA are shown.

Dysregulation of histone acetylation by HDAC inhibition has previously been shown to affect alternative RNA splicing (18,21). We found downregulation of RNA splicing factors in VPA-treated neurons. Therefore, we sought to determine whether global changes in alternative splicing events were associated with VPA exposure. We conducted replicate multivariate analysis of transcript splicing (rMATS) (39) and detected extensive changes in alternative splicing events in the VPA-treated samples after filtering out events with low exonic counts (<20) and events that showed less than a 10% change (Fig. 2A and Supplementary Material, Table S5). Briefly, we used three RNA-seq replicates for control and VPA-treated samples as input in the rMATS, and from these three replicates, the rMATS software detected statistically significant changes in alternative splice site usage in the VPA-treated samples versus the control samples. We report the number of statistically significant inclusion events for control and VPA samples. Skipped exon (SE) and retained intron (RI) events were the most frequent splicing events detected in VPA-treated neurons at 40 and 22% of total events, respectively (Fig. 2B). Other splice events observed were alternative 5′ or 3′ splice site (A5SS or A3SS) and mutually exclusive exon (Fig. 2B). Interestingly, VPA treatment led to decreased alternative exon inclusion in four out of the five different splice event types, suggesting that histone hyperacetylation could be implicated in the regulation of alternative splicing (Fig. 2B). For the purpose of illustrating associated gene changes, we show analysis of two genes *CLASP1* and *CEP170* which were among the most significantly altered in terms of differential splicing. Sashimi plots of representative examples for SE and RI, *CLASP1* and *CEP170*, respectively, showed fewer reads in alternatively spliced exons and greater skipped junction reads in VPA-treated samples (Fig. 2C and D).

### Alterations in transcript usage are observed primarily among genes associated with either RNA splicing or with neurodevelopmental disorders

**Figure 3:**
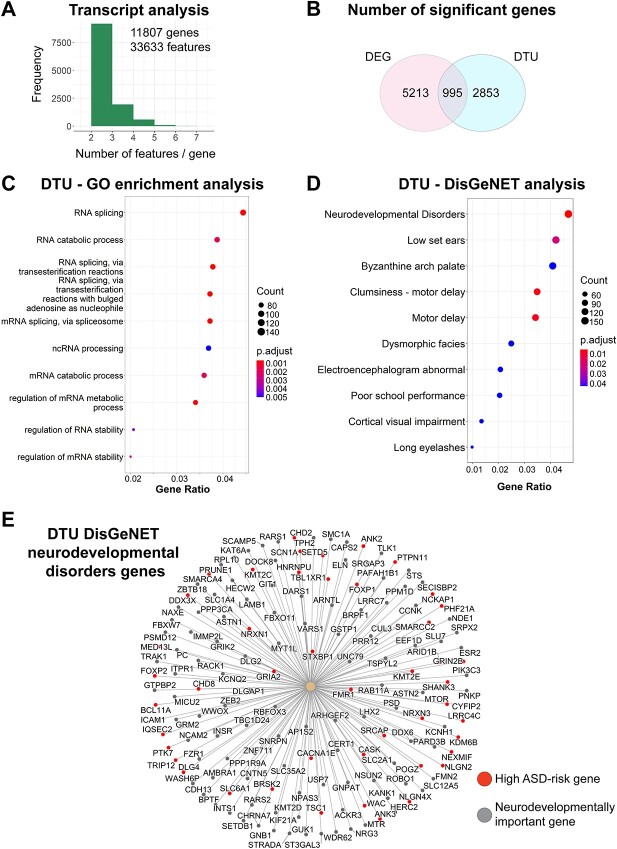
DTU analysis reveals DTU of genes associated with RNA splicing mechanisms and overrepresentation of genes associated with neurodevelopmental disorders. (**A**) Bar plot showing total number of genes and transcript isoforms analyzed in DRIMSeq DTU analysis in VPA and control samples. (**B**) Venn diagram showing overlap between significant DEGs (LFC > |1.5| and adjusted *P* < 0.05) and significant DTU events (*P* < 0.05). (**C**) GO analysis of significant DTU events (*q* value cutoff = 0.05). Top 10 enriched pathways are shown. (**D**) Characterization of disease pathways by DisGeNET analysis of significant DTU events. Top 10 enriched pathways are shown. (**E**) Network plot of genes showing significant DTU events identified in the neurodevelopmental disorder DisGeNET pathway. High confidence ASD risk genes (category 1 in SFARI gene database) are labeled in red. Neurodevelopmentally important genes are labeled in gray.

Differential RNA alternative splicing could have diverse phenotypic effects in the cell due to changes in frequency of non-coding RNAs, mRNA stability as well as gain or loss of protein domains, all which have the potential to alter cellular function and lead to disease (40). Through DRIMSeq DTU analysis (41), we analyzed a total of 11 807 genes with a total of 33 633 transcript isoforms (Fig. 3A and Supplementary Material, Table S6). We detected extensive changes in DTU events in VPA-treated neurons. Significant DTU events are defined with respect to changes in the proportions of transcript isoforms for a given gene. Therefore, we sought to define if these changes in transcript isoform expression in VPA-treated neurons compared with control neurons was occurring primarily in the DEGs. We determined the overlap between DEGs with genes that display significant DTU events (Fig. 3B). While we found an overlap of 995 DEGs that have DTU, our analysis showed that the majority of DTU (2853) was not occurring in DEGs. To begin to interrogate the extent to which the DTU genes might be different from the DEGs, we first conducted gene ontology (GO) biological process analysis on the DTU specific genes (Supplementary Material, Table S7). Interestingly, we identified an overrepresentation of genes involved in RNA biology which included RNA splicing, regulation of RNA stability and regulation of RNA metabolism (Fig. 3C). Next, we characterized the correlation of DTU with specific disease pathways using DISGeneNet databases (42) (Supplementary Material, Table S8). This analysis showed neurodevelopmental disorders as one of the largest and most statistically significant categories (Fig. 3D). However, the other disease groups, such as facial dysmorphisms, motor delay, cognitive problems or seizures (abnormal encephalogram), have also been associated multiple neurodevelopmental disorders and syndromic ASD (43). To further understand the molecular signatures associated with DTU-related neurodevelopmental disorders, we examined the genes in this category (Fig. 3E). We found an enrichment of genes that have high risk variants associated with ASD (i.e. *CHD8*, *FOXP1*, *SHANK3*) (44) and broader neuronal development (i.e. *ROBO1*, *NDE1*, *RACK1*) (45–48). Taken together, our work suggests that regulation of DTU might be essential for neurodevelopment.

### VPA differentially regulates gene expression and transcript usage of ASD-risk genes

**Figure 4:**
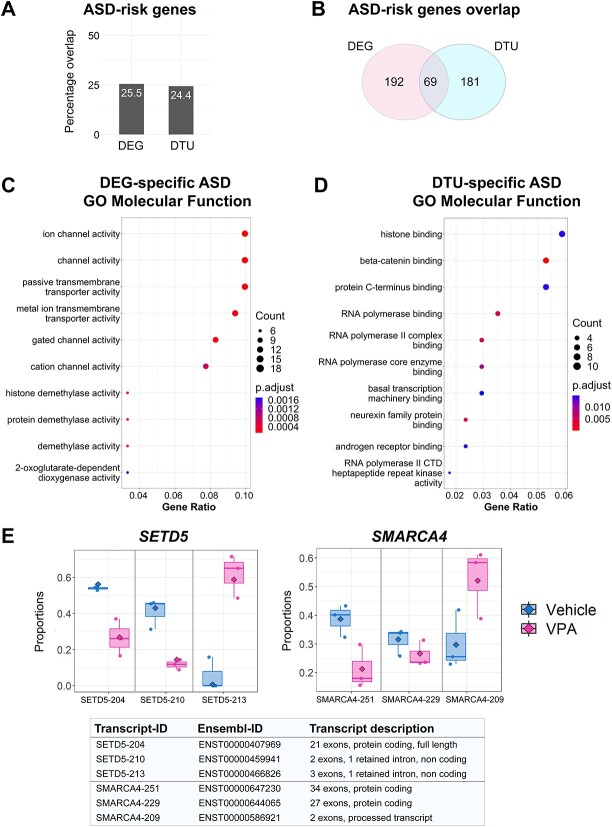
ASD-risk genes are differentially affected by gene expression and transcript usage in VPA-treated neurons. (**A**) Bar plot showing overlap between significant DEGs (FC ≥ |1.5| and adjusted *P* < 0.05) or DTU events (*P* < 0.05) with ASD-risk genes. (**B**) Venn diagram showing overlap of ASD-risk genes between significant DEGs and significant DTU events. (**C**) GO-molecular function analysis of DEG-specific ASD-risk genes. (**D**) GO-molecular function analysis of DTU-specific ASD-risk genes. (**E**) Analysis of two representative genes with DTUs. Box plots are shown for different transcripts in chromatin regulators *SETD5* (left panel) and *SMARCA4* (right panel). Transcripts found in vehicle treated neurons are shown in blue, and transcripts found in VPA treated neurons are shown in magenta. Description of different transcripts and ensemble IDs are shown in bottom panel.

The identification of ASD-risk genes among genes with DTU prompted us to define the extent to which ASD-risk genes overlapped with DEGs and DTU genes in VPA-treated neurons. We found that close to 25% of either DEGs or DTU genes were ASD-risk factors (Fig. 4A). However, only one-fourth of the ASD-risk genes among the DEGs and DTU genes overlapped (Fig. 4B). We performed GO analysis to determine whether the DEG- or DTU-specific genes in the ASD-risk gene group were enriched for different pathways (Supplementary Material, Table S9). Top pathways revealed from GO-biological process analysis of both DEG- and DTU-specific genes included: modulation of chemical synaptic transmission, regulation of post-synaptic signaling and synapse organization (Supplementary Material, Fig. S4). GO-cellular component analysis of both DEG- and DTU-specific genes revealed enrichment in synaptic membrane and pre-synapse pathways (Supplementary Material, Fig. S4). Interestingly, GO-molecular function analysis revealed that DEG- and DTU-specific genes were enriched in different molecular function pathways. DEG-specific genes were enriched for ion channel activity and transmembrane transporter activity (Fig. 4C). However, DTU-specific genes were enriched for histone binding, RNA II polymerase binding and transcription machinery binding (Fig. 4D).

To further define whether upregulated and downregulated ASD-specific DEGs were differentially enriched, we conducted GO-enrichment analysis across molecular function, cellular component and biological processes. GO-molecular function analysis of upregulated DEGs showed an overrepresentation of genes associated synaptic function (i.e. ion channel activity, calcium ion transporter activity). In contrast, GO-molecular function analysis of downregulated DEGs showed an enrichment in genes related to energy metabolism (i.e. dioxygenase activity, oxidoreductase activity) and protein methylation (i.e. histone methyltransferases and demethylases) (Supplementary Material, Fig. S4). GO-biological processes analysis of upregulated DEGs shows an overrepresentation of genes associated with synaptic transmission and organization, while downregulated DEGs showed an enrichment of genes involved in protein/histone methylation and demethylation as well as genes involved in the control of morphogenesis (Supplementary Material, Fig. S4). Finally, analysis of GO-cellular compartment of upregulated genes showed an overrepresentation of genes regulating synaptic structure and function (Supplementary Material, Fig. S4). However, we found no enrichment among the downregulated genes in the GO-cellular component analysis.

We then performed DisGeNET analysis on DEG- and DTU-ASD-specific risk genes and observed that both subsets were enriched for disease pathways such as neurodevelopmental disorders, severe intellectual disability, autistic behaviors and dysmorphic features (Supplementary Material, Fig. S5A and B). These pathways are concordant with co-morbidities present in syndromic autism. Furthermore, overlapping DEG- and DTU-ASD-specific risk genes showed similar enrichment (Supplementary Material, Fig. S5C). We also performed GO analysis on the overlapping ASD-risk genes and determined that these genes were enriched for pathways such as histone modification, chromatin remodeling and postsynaptic density (Supplementary Material, Fig. S5D and E).

We further analyzed how DTU was altering specific genes, by focusing on two representative ASD-risk genes *SETD5* and *SMARCA4* (Fig. 4E). Analysis of *SETD5* transcripts showed that VPA treated neurons had decreased levels of a full-length protein coding isoform and increased levels of one non-coding isoform (SETD5-213) but decreased expression of a different non-coding isoform (SETD5-210). Similarly, analysis of *SMARCA4* transcripts in VPA-treated neurons showed increased levels of the two-exon processed transcript (SMARCA4-209) but decreased levels of the longest protein coding isoform (SMARCA4-251) and a shorter protein coding isoform (SMARCA4-229). In summary, our analysis of ASD-risk genes dysregulated in VPA-treated neurons suggests that DEGs are enriched with genes associated with the control of synaptic organization and function, while genes with DTU are enriched with genes associated with control of transcription and chromatin architecture.

## Discussion

A recent study on non-mature human forebrain organoids treated with VPA for 3 days showed primarily alterations in synaptic transmission and ASD-risk genes which correlates with our own findings (49). However, in our study, we find that these same genes, in addition to other genes encoding chromatin, transcription and splicing regulators, display DTU, which may amplify VPA’s effect on gene expression. Previously, a transcriptomic study in rats showed that there were significant genome-wide changes in differential splicing in response to VPA (50). Similarly, gene-specific changes in differential exon usage have been reported for *BDNF* in mice treated with VPA in utero (51). We also find that VPA elicits differential gene expression of genes associated with RNA splicing, as well as genome-wide changes in RNA splicing, which is a unique signature that had not been previously reported in human neurons in response to VPA. Taken together, these data suggest that the effect of VPA in neuronal development might be mediated by changes not only on gene expression, but potentially in the control of alternative splicing mechanisms.

**Figure 5:**
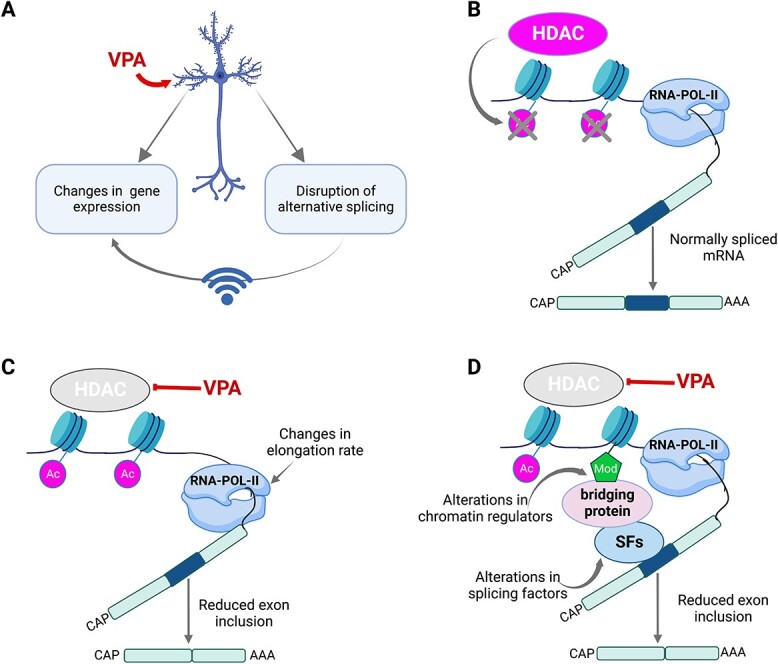
Potential models of VPA-induced changes in alternative splicing and DTU in human neurons. (**A**) Summary on the effect of VPA in human neurons. VPA elicits changes in gene expression and mRNA splicing. The effect of VPA in mRNA splicing could in turn further exacerbate VPA’s effect in the changes of gene expression. (**B**) Normal HDAC function, and the coupling of transcription by RNA polymerase II (RNA pol-II) and pre-mRNA splicing is shown. (**C**) Diagram shows changes in RNA elongation rate influenced by increased histone acetylation in VPA-treated neurons leading to defects in pre-mRNA splicing. (**D**) Diagram shows that alterations VPA exposure could lead to alterations in the splicing machinery or chromatin regulators that constitute interacting hubs for splicing factors, leading to alternative splicing defects.

The post-translational acetylation of histones is a well-known mechanism associated with the activation of transcription (52). Treatment with VPA, a potent HDAC inhibitor, results in a hyperacetylated chromatin environment, which mitigates HDAC-dependent transcriptional repression (13,14). In addition, the regulation of histone acetylation provides a broad mechanism to regulate alternative splicing and these mechanisms have been described in neurons (53) and astrocytes (54). Different HDAC enzymes have been shown to interact with different splicing factors (22), and the activity of HDACs is proposed to influence splice site selection (21). Histone hyperacetylation has also been shown to disrupt physical interaction between splicing factors and chromatin. For instance, SF3B1, a component of the U2 snRNP, was shown to physically associate with chromatin and facilitate splice-site recognition (55). Interestingly, in HeLa cells, addition of VPA disrupts the association between SF3B1 and chromatin leading to decreased exon inclusion levels of *SF3B1*-enriched exons (55). Furthermore, changes in the chromatin environment may affect the rate of elongation of RNA polymerase II (RNAPII), which may subsequently affect alternative splicing decisions through a kinetic coupling model which describes how perturbing RNAPII elongation rate can influence alternative splicing choices (56). Therefore, we posit that by inducing a hyperacetylated chromatin environment through its HDAC inhibitory function, VPA is altering not just gene expression but also RNA splicing (Fig. 5). Support for this hypothesis is provided by the identification of a general downregulation in the types of splicing events as well as a unique molecular signature of genes with DTU in VPA-treated neurons. We observed reduced numbers of alternative exon inclusion events in the VPA-treated neurons. This finding coincides with the downregulation of *RBFOX2* and the presence of DTU in *RBFOX3* in response to VPA. RBFOX genes encode splicing regulatory factors that have been associated with either promoting or repressing exon inclusion during splicing (57). Therefore, the distinct dysregulation of both *RBFOX2* and *RBFOX3*, among other genes encoding splicing factors, could contribute in part to the overall alterations in RNA splicing associated with VPA exposure.

The influence of the chromatin environment in the co-transcriptional regulation of RNA splicing is achieved by numerous mechanisms that implicate chromatin remodeling and post-translational modifications of histones (58). Our work shows that expression of genes related to histone and chromatin regulators were significantly affected. In addition, we observed DTU in several chromatin modifiers and remodelers that, at least in the case of *SETD5* and *SMARCA4*, seem to preferentially downregulate their coding isoforms but upregulate their non-coding or truncated protein isoforms, which suggests an additional mechanism that would impair the function of different chromatin regulators. Taken together, the alterations in gene expression and transcript usage of chromatin regulatory proteins could additionally impact splicing outcomes in response to VPA. One well-studied example of a histone modification that regulates RNA splicing is methylation of H3K36, a highly conserved histone modification. In yeast, it was found that H3K36 methylation was required for proper co-transcriptional spliceosome assembly by recruiting a chromodomain protein that stabilized interaction between splicing factors and chromatin (59). In humans, H3K36me3 was implicated in regulating alternative splicing via recruitment of polypyrimidine tract-binding protein via chromodomain protein MRG15 (58). We find evidence that VPA modulates genes encoding histone methyltransferases that methylate H3K36, such as NSD1 (60) and SETD5 (61). NSD1 has been associated with syndromic ASD (62) and modulates the mono- and di-methylation of H3K36 (H3K36me1/2) (60). Human neurons treated with VPA showed significantly decreased expression of *NSD1* in this study. H3K36me2 serves as a substrate for the subsequent tri-methylation of H3K36. Therefore, loss of NSD1 could lead to a widespread reduction of H3K36me3 at a genome-wide scale, which in turn could disrupt alternative splicing. Similarly, we find that SETD5, which catalyzes the trimethylation of H3K36 (61), shows DTU with decreased levels of the protein coding isoform and either upregulation or downregulation of two different non-coding isoforms. Mice haploinsufficient for SETD5 have decrease neuronal progenitor proliferation, synaptic defects, decreased sociability and impairments on cognitive tasks, which correlates with loci-specific reduction of H3K36me3 (61), and increased chromatin hyperacetylation (63). Taken together, these data suggest that in human, neurons exposure to VPA could reduce the expression of *NSD1* and the coding *SETD5* isoform and lead to decreased H3K36me3 levels, which in turn can affect RNA splicing.

In addition to changes in H3K36me3, changes in H3K9 methylation have been suggested to affect alternative splicing. H3K9me3 is enriched on alternative exons and has been shown to recruit the chromodomain protein HP1γ to regulate alternative splicing by reducing the rate of RNAPII elongation (64). Similarly, H3K9me2 has been shown to regulate alternative splicing through recruitment of HP1α and subsequent reduction of the rate of RNAPII elongation (65). We found that expression of *SETDB1*, an ASD-risk gene and a histone methyltransferase involved in catalyzing H3K9 methylation, is significantly reduced in our VPA-treated neurons. Therefore, we posit that dysregulation of H3K36 and H3K9 methylation, as well as potentially dysregulation of other histone modifications (58,66), could alter the chromatin environment further exacerbating the alternative splicing defects associated with the hyperacetylation of chromatin caused by VPA exposure.

The relevance of our findings on the dysregulation of alternative splicing to the mechanisms that underlie ASD pathogenesis is suggested by its correlation with previous transcriptomic studies of ASD-postmortem brain tissue. A study of over 1600 post-mortem cerebral cortex samples from ASD, schizophrenia (SCZ) and bipolar disorder (BP) individuals showed that across the three disorders changes in isoform-level had the largest effect size associated with disease (67). Moreover, like our own findings in which VPA induces enrichment of splicing regulatory proteins among genes with DTU, analysis of ASD, SCZ and BP cerebral cortices also showed disease association in the splicing of RNA binding proteins and splicing factors (67). Furthermore, a study of 48 ASD-postmortem cerebral cortex tissue showed downregulation of genes involved in the control of alternative splicing (33). We present three possible mechanisms by which chromatin dysregulation by VPA affects RNA splicing in human neurons that could potentially contribute to ASD pathogenesis (Fig. 5). However, additional studies will be necessary to precisely dissect the contribution of the different mechanisms that underlie how VPA can alter RNA splicing. In summary, our work highlights the importance of the chromatin environment in the regulation of alternative splicing in the pathogenesis of ASD associated with environmental risk factors and potentially genetic risk factors (chromatin regulators).

## Materials and Methods

### Induced pluripotent stem cell culture

We used a control iPSC line I-93-7 obtained from a healthy neurotypical male individual that was previously characterized (36). Human iPSCs were cultured as previously described (36,68). Briefly, cells were cultured using a feeder-free system and mTeSR media (STEMCELL technologies catalog # 85850). Cultures were inspected daily for spontaneous differentiating colonies which were manually removed to ensure the purity of the stem cell cultures. iPSCs were passaged every 4–7 days with ReLeSR reagent (STEMCELL technologies catalog #100-0484) at a ratio of 1:6 per culture. In order to ensure that no mutations were introduced in the iPSC cultures, we used cultures that were passaged for less than 40 cycles.

### Neuronal differentiation and VPA treatment

Human control iPSCs were differentiated using a previously described dual SMAD inhibition protocol that promotes the formation of the neuroectoderm by preventing the formation of the mesoderm and endoderm by targeting the BMP signaling pathway (30). This protocol primarily produces excitatory forebrain cortical neurons in a monolayer and was conducted as previously described (36). In brief, human iPSCs were dissociated into single cells using Gentle Cell Dissociation Reagent (STEMCELL Technologies catalog # 100-0485) and were grown for 1 day with STEMdiff Neuronal induction medium (STEMCELL Technologies catalog # 05835), supplemented with 10 μM ROCK inhibitor (Y-27632 dihydrochloride, TOCRIS catalog # 1254). After a confluent monolayer formed, cells were switched to a neuronal induction medium containing two SMAD inhibitors: 1 μM Dorsomorphin (STEMGENT catalog # 04-0024) and 10 μM SB 431542 (TOCRIS catalog # 1614), and all subsequent steps were conducted as previously described (30,36). After day 45 of neuronal induction, cultures were supplemented with 10 ng/μl laminin every other day to ensure they remained attached. At day 64, three independent cultures were treated with 200 μg/ml VPA or DMSO at day 64 of neuronal induction and were harvested after 24 h for RNA extraction.

### Library preparation and sequencing

To analyze changes in the neuronal transcriptome, cells were harvested on ice, and RNA was extracted on the same day using mirVANA RNA isolation kit (Thermofisher catalog # 1560) per manufacturer’s guidelines. RNA quality was assessed using a Bioanalyzer (Supplementary Material, Fig. S1A and B). Samples with RNA integrity number higher than 9 were used for RNA sequencing. cDNA libraries were prepared using the Illumina Tru-Seq kit (Illumina catalog # FC-122-1001). Isolated cDNA libraries were sequenced by paired-end chemistry via Illumina HiSeq 2500. On average, 300 millions of 50 bp paired-end reads were obtained from each library.

### RNA-seq data processing and analysis

The quality of raw sequence data in FastQ files generated following sequencing was accessed using FastQC (version 0.11.9) (69) (Supplementary Material, Fig. S1C). Raw reads were mapped to the Ensembl reference genome 104 (GRCh38) using the transcript-level quantifier Salmon (version 1.5.2) in mapping-based mode (37). An average of 149 416 071.5 fragments were observed with an average mapping rate of 82.96% (Supplementary Material, Table S1). The tximport software package (version 1.20.0) was used to produce count matrices from the transcript-level quantification files produced by Salmon (70). Following transcript-to-gene mapping, DEGs were detected using the DESeq2 software package (version 1.32.0) (71). DEGs were identified as statistically significant if they had an adjusted *P*-value of <0.05 and a fold change ≥ |1.5| (Supplementary Material, Table S2). Pathway enrichment analysis was conducted using gene datasets available through GSEA using the GSEA algorithm on a ranked list (signed fold change * −log_10_  *P*-value) of DEGs in the ClusterProfiler package in R (72). The gene datasets used (33 196 gene sets) are found in the Human Molecular Signatures Database (MSigDB).

### Differential alternative mRNA splicing and transcript usage analysis

DTU events were identified using DRIMSeq software package (version 1.20.0) (41). Lowly expressed transcripts were filtered from analysis. Transcripts needed to have at least 10 estimated counts in at least 3 samples. Additionally, transcripts needed to have a minimum proportion of 0.1 in at least 3 samples. Statistically significant DTU events were identified following stageR (version 1.14.0) post-processing analysis as events that has an adjusted *P* < 0.05. Differential alternative splicing events were identified using rMATS (version 4.1.1) (39). Statistically significant differential alternative splicing events were identified as events with FDR < 0.05 and IncLevelDifference ≥ |0.1|. Exons with total read counts ≤ 20 were excluded from the analysis. ClusterProfiler (version 4.0.5) was used for functional enrichment analysis (GSEA, GO and DisGeNET) of DEGs and DTU events. Significant enrichment results were obtained using a *q*-value cutoff of <0.05.

### Validation of RNA-seq by qPCR

Differential gene expression for a subset of genes was validated using qPCR as previously described (68). Primers were purchased in IDT (Supplementary Material, Table S3).

## Supplementary Material

Leung_etal_Sup_Material-HMG-2002-CE-00739_ddad002

Leung_etal_Sup_Tables-S1-S9_ddad002

## Data Availability

The RNA-sequencing data reported in this paper are uploaded in Gene Expression Omnibus (GEO) under accession number GEO: GSE222509.
