# Supplementary material for: Dysregulation of the chromatin environment leads to differential alternative splicing as a mechanism of disease in a human model of autism spectrum disorder
Source: Hum Mol Genet. 2023 Jan 9;32(10):1634–46. doi: 10.1093/hmg/ddad002 (PMC10162432; doi:10.1093/hmg/ddad002)
Supplement: Leung_etal_Sup_Material-HMG-2002-CE-00739_ddad002 [file leung_etal_sup_material-hmg-2002-ce-00739_ddad002.docx]

**DYSREGULATION OF THE CHROMATIN ENVIRONMENT LEADS TO DIFFERENTIAL ALTERNATIVE SPLICING AS A MECHANISM OF DISEASE IN A HUMAN MODEL OF AUTISM SPECTRUM DISORDER**

Calvin S. Leung ^1, 2^, Shoshanna Rosenzweig ^3, 4, 5^, Brian Yoon ^6^, Nicholas A. Marinelli ^6^, Ethan W. Hollingsworth ^7, 8^, Abbie M. Maguire ^1,2^, Mara H. Cowen ^6^, Michael Schmidt ^1,2^, Jaime Imitola ^7,8^, Ece D. Gamsiz Uzun ^3. 4, 5,*^ , and Sofia B. Lizarraga^1, 2, *^

**Supplementary Material**

^1^ Department of Molecular Biology, Cell Biology and Biochemistry, Brown University, Providence, RI 02912, USA

^2^ Center for Translational Neuroscience, Carney Institute for Brain Science and Brown Institute for Translational Science (BITS), Brown University, Providence, RI 02912, USA

^3^ Center for Computational Molecular Biology, Brown University, Providence, RI 02906

^4^ Department of Pathology and Laboratory Medicine, Warren Alpert Medical School of Brown University, Providence, RI 02912

^5^ Department of Pathology and Laboratory Medicine, Rhode Island Hospital and Lifespan Academic Medical Center, Providence, RI 02903

^6^ Department of Biological Sciences, University of South Carolina, 715 Sumter Street, Columbia, SC 29208

^7^ UCONN Health Comprehensive Multiple Sclerosis Center, Department of Neurology, University of Connecticut School of Medicine, Farmington, CT 06030

^8^ Division of Multiple Sclerosis and Translational Neuroimmunology, Department of Neurology, University of Connecticut School of Medicine, Farmington, CT 06030

^*^ Co-corresponding authors

[sofia_lizarraga@brown.edu](mailto:sofia_lizarraga@brown.edu)
70 Ship St Rm 323, Providence, RI 02903

Phone: 401- 863- 1692

[dilber_gamsiz@brown.edu](mailto:dilber_gamsiz@brown.edu)
Phone: 401-793-8190

**SUPPLEMENTARY FIGURES AND FIGURE LEGENDS**

**
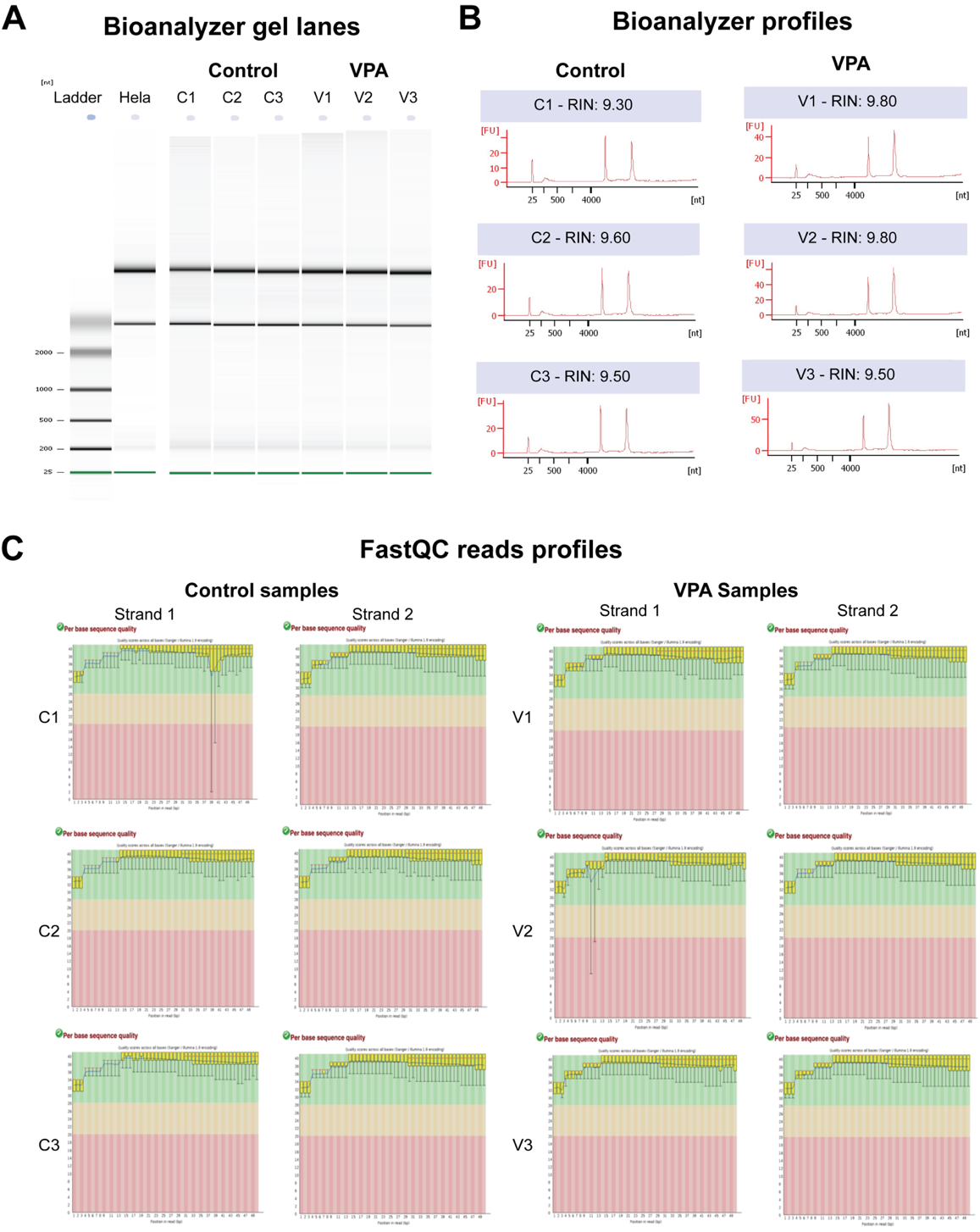
Supplementary Figure S1**

**Supplementary Figure S1: Quality control for total RNA and RNA-seq reads. (A-B)** Analysis of total RNA quality by bioanalyzer. RNA bands (**A**) as well as the bioanalyzer profiles with RNA integrity number (RIN) (**B**) are shown for all control (vehicle) and VPA treated samples. (**C**) Fast-QC profiles show the quality of the RNA-reads in each RNA library for both strands in each control or VPA treated sample.

**
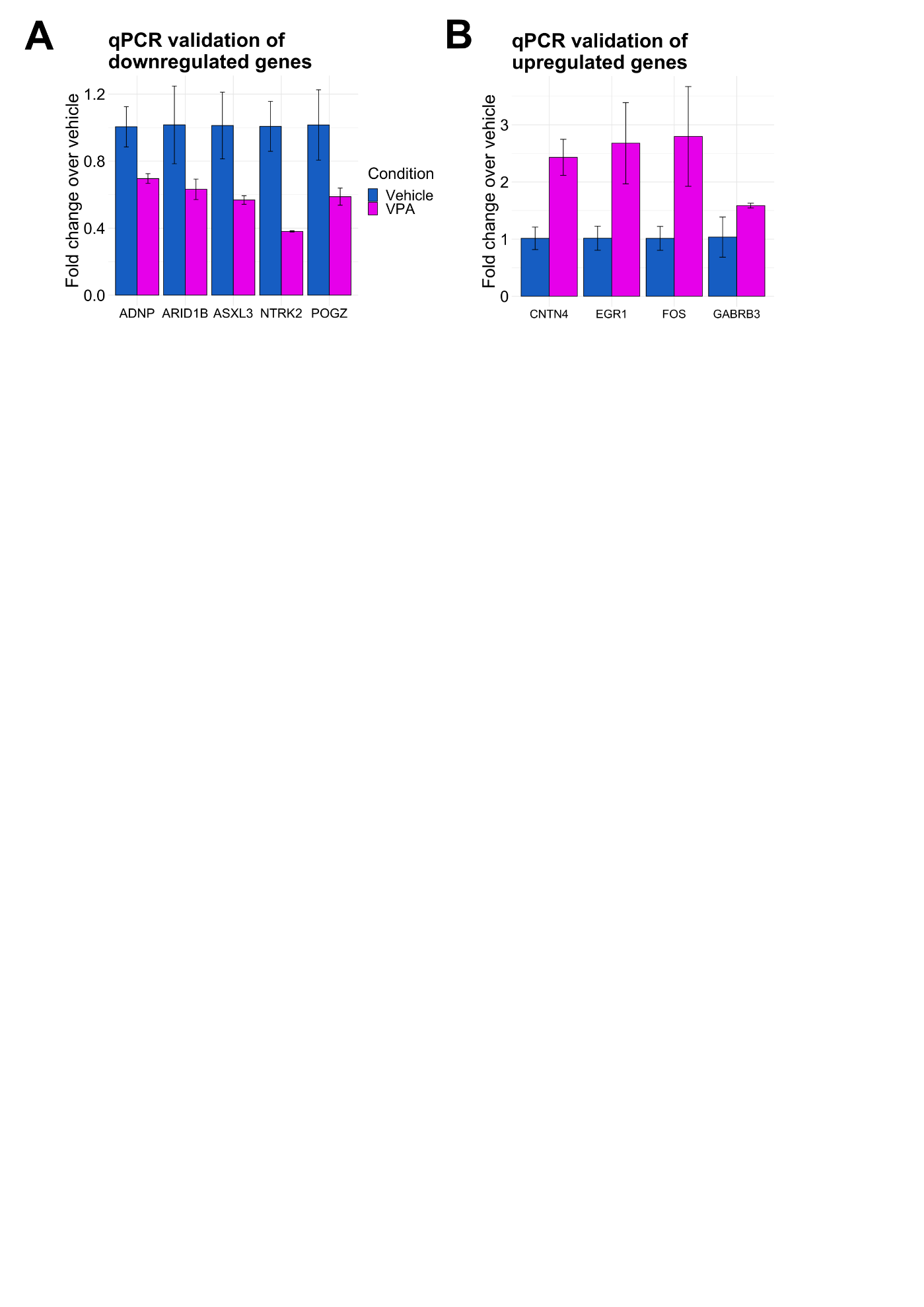
Supplementary Figure S2**

**Supplementary Figure S2: Gene expression validation of DESeq2 results. (A)** Gene expression analysis by qPCR of representative significantly downregulated DEGs in vehicle (blue) and VPA (magenta) treated neurons. **(B)** Gene expression analysis by qPCR of representative significantly upregulated DEGs, in vehicle (blue) and VPA treated neurons. Error bars represent standard deviation.

**
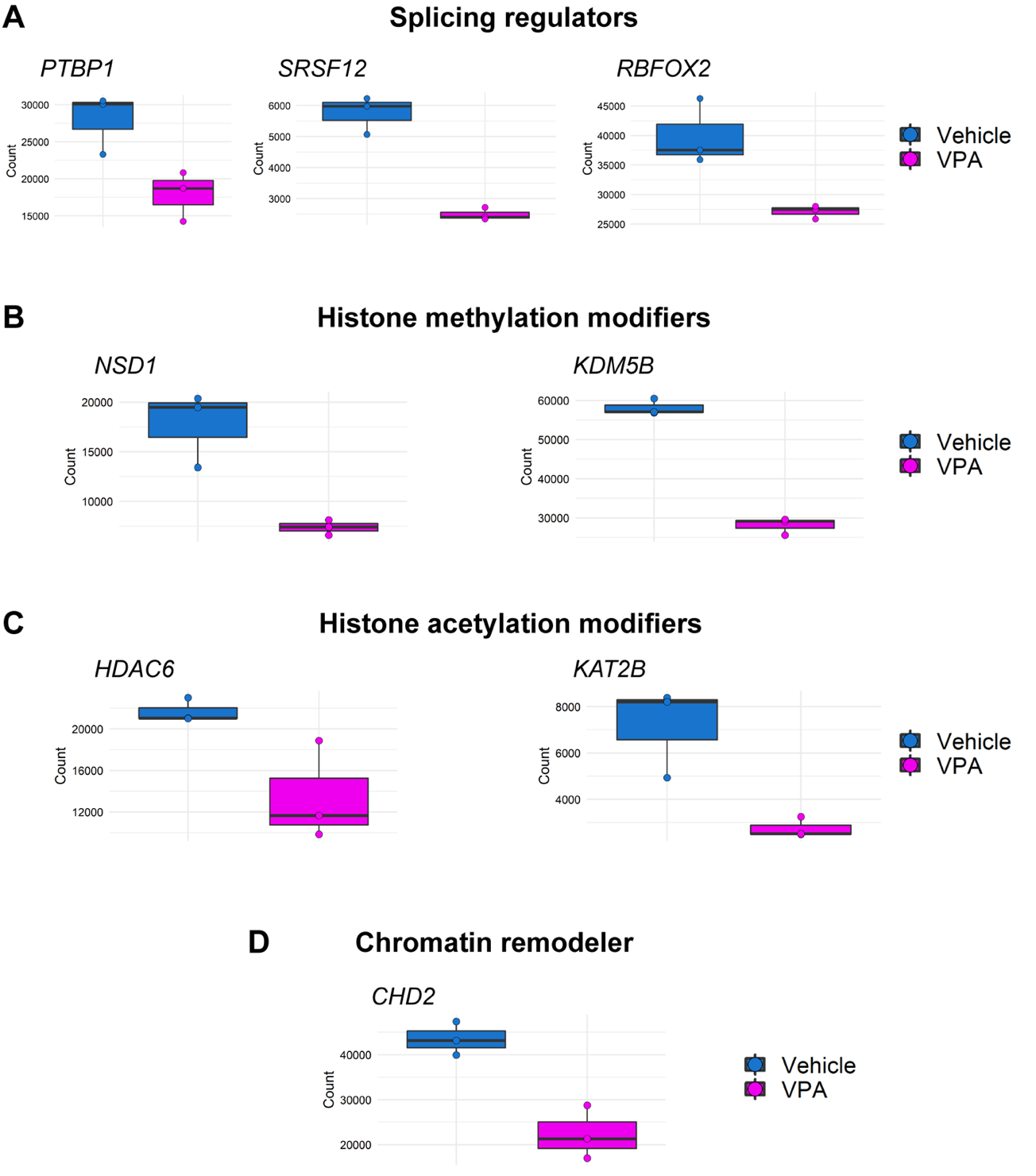
Supplementary Figure S3**

**Supplementary Figure S3: Normalized DESeq2 gene counts of genes identified in GSEA analysis. (A)** Normalized gene counts of representative alternative splicing factors identified in the regulation mRNA processing pathway in vehicle (blue) and VPA (magenta) samples. **(B)** Normalized gene counts of representative genes identified in the covalent chromatin modification, histone modification, and DNA conformation change pathways associated with the control of histone methylation in vehicle (blue) and VPA (magenta) samples. **(C)** Normalized gene counts of representative genes identified in the covalent chromatin modification, histone modification, and DNA conformation change pathways associated with the control of histone acetylation in vehicle (blue) and VPA (magenta) samples. **(D)** Normalized gene counts of representative genes identified in the covalent chromatin modification, histone modification, and DNA conformation change pathways associated with the control of chromatin remodeling in vehicle (blue) and VPA (magenta) samples. Error bars represent standard deviation.


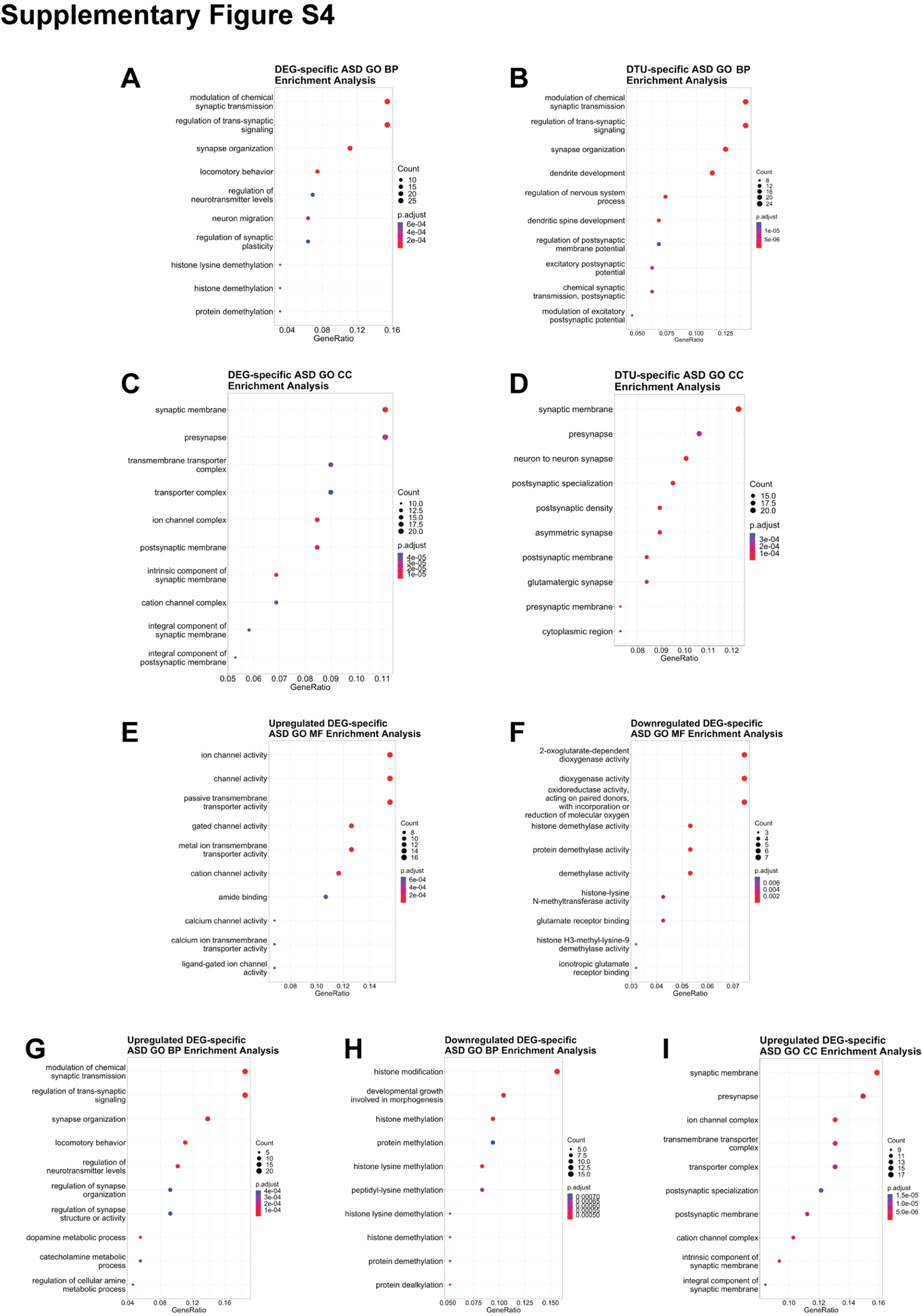
**Supplementary Figures S4**

**Supplementary Figure S4: Extended gene ontology (GO) analysis of DEG- and DTU-specific ASD-risk genes and dissection of upregulated and downregulated DEG GO analysis. (A-B)** GO Biological Processes (BP) analysis of DEG-specific ASD-risk genes (**A**) and of DTU-specific ASD-risk genes (**B**). Top 10 enrichment pathways are shown for each. *P* value cutoff = 0.05. (**C-D)** GO Cellular component (CC) analysis of DEG-specific ASD-risk genes (**C**) and of DTU-specific ASD-risk genes (**D**). Top 10 enrichment pathways are shown. *P* value cutoff = 0.05. (**E-F)** GO Molecular function (MF) analysis of upregulated (**E**) and downregulated (**F**) DEG-specific ASD-risk genes. Top 10 enrichment pathways are shown for each. *P* value cutoff = 0.05. (**G-H)** GO BP analysis of upregulated (**G**) and downregulated (**H**) DEG-specific ASD-risk genes. Top 10 enrichment pathways are shown for each. *P* value cutoff = 0.05. (**I)** GO CC analysis of upregulated DEG-specific ASD-risk genes. Top 10 enrichment pathways are shown for each. *P* value cutoff = 0.05.


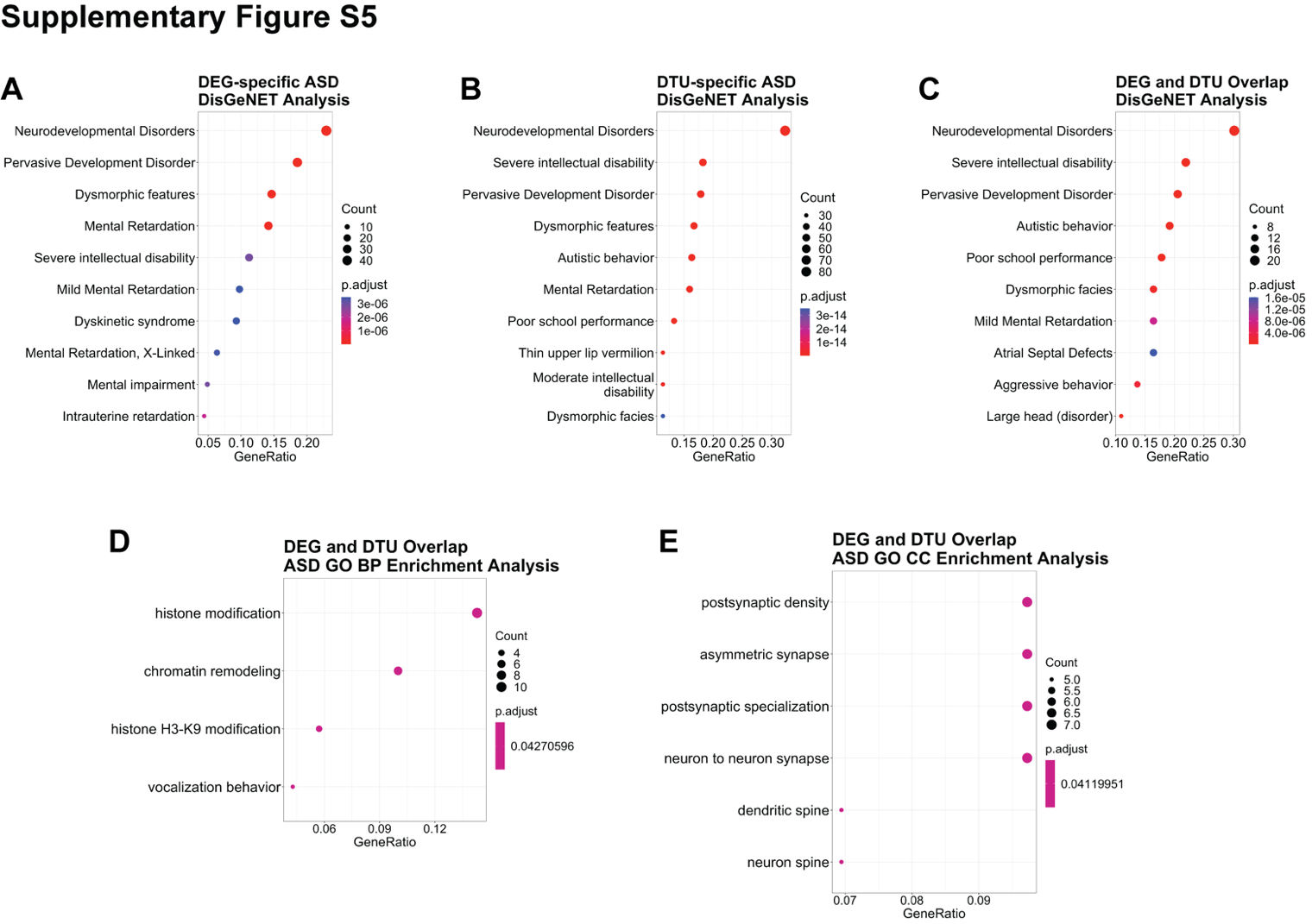
**Supplementary Figure S5**

**Supplementary Figure S5: DisGeNET analysis of DEG- and DTU-ASD-specific risk genes and analysis of the overlapping DEG and DTU -ASD-specific risk genes by DisGeNET and GO analysis. (A-B)** DisGeNET analysis of DEG-specific ASD-risk genes (**A**) and of DTU-specific ASD-risk genes (**B**). Top 10 enrichment pathways are shown for each. *P* value cutoff = 0.05. (**C**) DisGeNET analysis of the overlap between DEG- and DTU-specific ASD-risk genes. Top 10 enrichment pathways are shown. *P* value cutoff = 0.05. (**D)** GO Biological Process (BP) analysis of the overlap between DEG- and DTU-specific ASD-risk genes. Top 10 enrichment pathways are shown for each. *P* value cutoff = 0.05. (**E)** GO Cellular Component (CC) analysis of the overlap between DEG- and DTU-specific ASD-risk genes. Top 10 enrichment pathways are shown for each. *P* value cutoff = 0.05.

**LEGENDS FOR SUPPLEMENTARY TABLES**

All supplementary tables are in an excel format in dataset1.

**Supplementary Table S1: Information on Sequencing Reads.** Table shows the total number of fragments analyzed and the percentage of mapping for each sample.

**Supplementary Table S2: Differential gene expression analysis.** Table shows downregulated and upregulated DEGs detected with DESeq2 software package (version 1.32.0) ^1^ using an adjusted *P* < 0.05 and a fold change ≥ |1.5|. Fold change is shown in logarithmic scale, the standard error estimate for the log2 gold change is shown as lfcSE, the regular and adjusted P values are also shown.

**Supplementary Table S3: Primers for qPCR analysis.** Table shows the primers used for gene expression analysis. Primers were pre-designed to cover all isoforms for each gene. Forward and Reverse primer sequences are shown.

**Supplementary Table S4: Gene Set Enrichment Analysis.** Table shows all the categories identified by Gene Set Enrichment Analysis (GSEA) database. The total number of genes in each category are shown in the setSize column. Gene ontology identification numbers (ID) are shown for each category and the genes included in each category are shown under the core_enrichment column and are listed by their Ensembl IDs. The normalized enrichment score (NES) is calculated by GSEA and is corrected for multiple hypothesis testing (FDR). The upregulated categories have a positive enrichment score or NES and the downregulated categories have a negative enrichment score or NES.

**Supplementary Table S5: Analysis of alternative splicing events using rMATs.** Separate tables are shown for each type of alternative splicing event. The events analyzed include: Skipped exon (SE), retained intron (RI), alternative 5’ or 3′ splice site (A5SS or A3SS) and mutually exclusive exon (MXE). Genes are shown by gene symbol, with annotations for: chromosome, strand orientation, exon start (ES), exon end (EE) locations, false discovery rate (FDR), and inclusion level (IncLevel) is shown as well as the inclusion level difference (IncLevelDifference).

**Supplementary Table S6: Differential transcript usage analysis using DRIMseq.** Table shows all genes with DTU. The Gene column represents the FDR of whether that particular gene undergoes DTU. The Transcript column represents whether that particular transcript of the gene shows differential expression levels compared to other transcripts of that same gene.

**Supplementary Table S7: Gene ontology analysis of significant DTU events.** Table shows gene ontology analysis for genes with DTU events. The gene ontology (GO) categories analyzed were molecular function (MF), biological process (BP), and cellular compartment (CC) for all the different genes. GeneRatio represents the number of significant genes found in a GO term / total number of significant genes. BgRatio represents the number of genes in a GO term / total number of background genes. Genes for each category are shown by their Ensembl ID.

**Supplementary Table S8: Analysis of disease pathways across significant DTU events using DisGene databases.** Table shows analysis of disease pathways each pathway has a unique GO identifier. Genes in each category are shown by their gene symbol. Gene ratio and Bg ratio are shown along with normal and adjusted p values.

**Supplementary Table S9: Gene ontology analysis of ASD-risk genes with specific differential gene expression or differential transcript usage.** Tables show GO analysis for ASD-risk genes with either DEG (DEG-ASD) or DTU events (DTU-ASD). The gene ontology (GO) categories analyzed were molecular function (MF), biological process (BP), and cellular compartment (CC). GeneRatio represents the number of significant genes found in a GO term with respect to the total number of significant genes. BgRatio represents the number of genes in a GO term with respect to the total number of background genes. Genes for each category are shown by the Ensembl ID.

**REFERENCES**

1 Love, M. I., Huber, W. & Anders, S. Moderated estimation of fold change and dispersion for RNA-seq data with DESeq2. *Genome Biol* **15**, 550, doi:10.1186/s13059-014-0550-8 (2014).
